# Observational Review and Analysis of Concussion: a Method for Conducting a Standardized Video Analysis of Concussion in Rugby League

**DOI:** 10.1186/s40798-017-0093-0

**Published:** 2017-07-14

**Authors:** Andrew J. Gardner, Christopher R. Levi, Grant L. Iverson

**Affiliations:** 1 Centre for Stroke and Brain Injury, School of Medicine and Public Health, University of Newcastle, Callaghan, New South Wales Australia; 2 Hunter New England Local Health District Sports Concussion Program, John Hunter Hospital, Newcastle, New South Wales Australia; 3 Department of Physical Medicine and Rehabilitation, Harvard Medical School, Spaulding Rehabilitation Hospital, Boston, MA USA; 4 MassGeneral Hospital for Children™ Sport Concussion Program, Boston, MA USA; 5 Home Base, A Red Sox Foundation and Massachusetts General Hospital Program, Boston, MA USA

**Keywords:** Concussion, Video analysis, Injury management

## Abstract

**Background:**

Several professional contact and collision sports have recently introduced the use of sideline video review for club medical staff to help identify and manage concussions. As such, reviewing video footage on the sideline has become increasingly relied upon to assist with improving the identification of possible injury. However, as yet, a standardized method for reviewing such video footage in rugby league has not been published. The aim of this study is to evaluate whether independent raters reliably agreed on the injury characterization when using a standardized observational instrument to record video footage of National Rugby League (NRL) concussions.

**Methods:**

Video footage of 25 concussions were randomly selected from a pool of 80 medically diagnosed concussions from the 2013–2014 NRL seasons. Four raters (two naïve and two expert) independently viewed video footage of 25 NRL concussions and completed the Observational Review and Analysis of Concussion form for the purpose of this inter-rater reliability study. The inter-rater reliability was calculated using Cohen’s kappa (*κ*) and intra-class correlation (ICC) statistics. The two naïve raters and the two expert raters were compared with one another separately.

**Results:**

A considerable number of components for the naïve and expert raters had almost perfect agreement (*κ* or ICC value ≥ 0.9), 9 of 22 (41%) components for naïve raters and 21 of 22 (95%) components for expert raters. For the concussion signs, however, the majority of the rating agreement was moderate (*κ* value 0.6–0.79); both the naïve and expert raters had 4 of 6 (67%) concussion signs with moderate agreement. The most difficult concussion sign to achieve agreement on was blank or vacant stare, which had weak (*κ* value 0.4–0.59) agreement for both naïve and expert raters.

**Conclusions:**

There appears to be value in expert raters, but less value for naive raters, in using the new Observational Review and Analysis of Concussion (ORAC) Form. The ORAC Form has high inter-rater agreement for most data elements, and it can be used by expert raters evaluating video footage of possible concussion in the NRL.

## Key Points

- Identifying concussion from the sideline during a match is challenging, but with the use of video out-of-view or fleeting signs may be captured and a player can be removed from play.
- Having a reliable form for coding and analysing concussion can be a useful adjunct to the sideline clinical management strategy of the athletic trainer and team physician.

We present the first objective and reliable coding form for rugby league to capture the game situation, the mechanism of injury, and possible signs of concussion.

## Background

Rugby league is a high-intensity collision sport [23]. The game is played continuously in two 40-min halves, and game-play involves two teams of 13 on-field players and four interchange players who may be switched in and out of the game. The published incidence rates of concussion in rugby league vary [12]; at the National Rugby League (NRL) level, medically diagnosed concussions in three clubs from the 2013 season revealed an incidence rate of 14.8 concussions per 1000 player match hours [15], while a rate of 28.3 concussion per 1000 player match hours were reported from one NRL club over a 15-year (1998–2012) period [41]. The incidence of use of the concussion interchange rule (CIR) was 24.0 (95% CI 20.7–27.9) uses of the CIR per 1000 NRL player match hours [14] and 44.9 (95% CI 38.5–52.3) uses of the CIR per 1000 National Youth Competition player match hours [15].

One method that has becoming increasingly relied upon to assist with improving the identification of possible concussion has been the review of video footage on the sideline. The use of video for reviewing a concussion may identify signs of injury that may have been blocked from view or otherwise missed by medical staff. A number of professional contact and collision sports have recently introduced the use of sideline video review for club medical staff to help identify and manage concussions [29]. A number of studies of video footage have been conducted in a variety of sports, for example, rugby league [13–15], rugby union [25], and Australian Rules Football [9, 29, 30]. Other sports, such as boxing [37], soccer [1], taekwondo [24], ice hockey [6, 10, 18, 19], and lacrosse [28], have also reported on the use of video footage for understanding the circumstances and mechanisms of injury unique to their sports. A risk prediction model among National Hockey League (NHL) players reported that both visual signs of concussion and information pertaining to mechanisms of injury improved a clinician’s ability to identify athletes who should be removed from play and evaluated [6]. Specifically, the study indicated that suspected loss of consciousness, motor incoordination or balance problems, being in a fight, having an initial hit from another player’s shoulder, and having a secondary hit on the ice were all associated with increased risk of subsequent concussion diagnosis.

Sport-specific coding criteria of concussion for game situational factors and injury mechanisms have been developed for hockey (e.g., the ‘Heads-Up Checklist’ [20]), but these criteria do not generalize to other sports like rugby union or rugby league. Video criteria and coding forms require validation in each individual sport [29, 30]. In a more recent NHL study examining the predictive ability of visual signs of concussion, loss of consciousness, motor incoordination, and blank/vacant look had a positive association with concussion diagnosis, whereas slow to get up and clutching of the head, despite occurring frequently, had low positive predictive values [10].

Several video studies have examined signs of concussion, together with player characteristics, injury characteristics, and match situational factors, in professional rugby league [13–15]. In 2014, video reviews of injury have been implemented in the NRL to help medical staff and promote player health and safety. The aim of this study was to present a standardized observational recording form and to determine whether independent raters agreed on the antecedent events, mechanisms of injury, and concussion signs when using the form to code digital video records of concussions in the NRL.

## Methods

### Participants

This study was conducted in the national professional rugby league competition in Australia during the 2013 and 2014 seasons. All medically diagnosed concussion events during 2013 and 2014 NRL seasons were available to be included in this study.

### Procedure

For this study, 25 medically diagnosed concussions were randomly selected from the 2013 to 2014 NRL seasons’ video library (*n* = 80). The video library included only excerpts of the incidents for each case, not the entire game. The duration of the game footage recorded for each of the 25 cases selected from the library and used in this study ranged from 138 to 473 s. Four raters (two ‘expert’ and two ‘naïve’) independently reviewed the video footage of the 25 NRL concussion events. The naïve raters were novices of the sport. They had limited to no knowledge or experience with rugby league match play and no experience identifying and managing concussion. The expert raters were defined as individuals with experience in rugby league match play and expertise in concussion management at the professional (NRL) level. Both expert raters had at least one NRL season of experience working on the sideline for an NRL club with the responsibility of identifying and assessing athletes suspected of having sustained a concussion.

The medically diagnosed NRL concussion library was gathered from three teams during the 2013 season (*n* = 20 concussions) [13], and all teams during the 2014 NRL season (*n* = 60 concussions). The Concussion in Sport Group consensus definition of concussion was used by all clubs involved in this study [32]. Raters viewed the match digital records of 25 concussions using the Quicktime Multimedia Player V.7.7.5. Each rater completed all components of the form for each of the 25 concussion events. The raters were permitted to view the incident as many times as required and in any playback speed as deemed necessary to complete all categories of the form. All participants provided informed consent. This study was conducted in accordance with the standards of the ethics outlined in the Declaration of Helsinki. Approval of this study was provided by the University of Newcastle Human Ethics Committee.

### Instruments

**Fig. 1:**
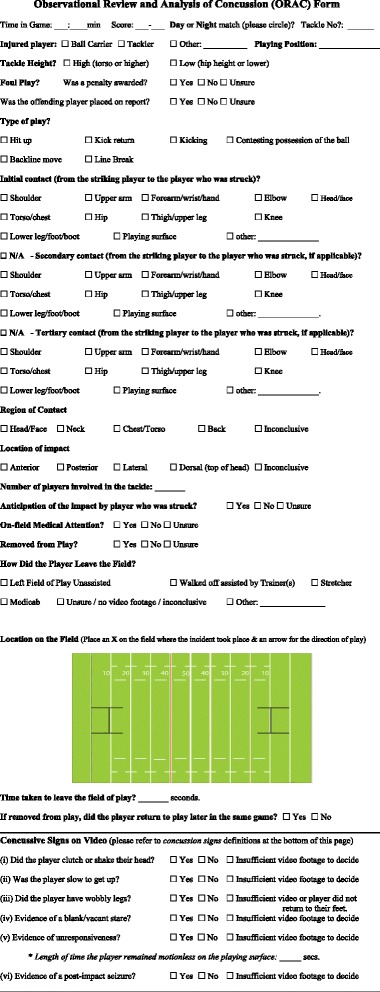
The Observational Review and Analysis of Concussion (ORAC) Form

A rating form was created to provide a simple but standardized framework for coding and analysing video footage of the situations and consequences of concussion events in rugby league. The form was developed by a neuropsychologist with extensive experience in the sport and concussion management and was based on work conducted previously in ice hockey in North America, and a similar, but not identical, approach to validating the form was used for the validation of the ‘Heads-Up Checklist’ [20]. The form was adapted to include specific information to rugby league. Concussion signs that have been previously examined in video review studies in rugby league were included [13–15]. The form consists of various sections related to the player and game characteristics (e.g., ball carrier versus tackler, tackle height, type of play, etc.), the anatomical region of contact, the injury location on the field of play, the injured player’s on-field management, and six possible concussion signs (see Fig. 1).

### Statistical Analysis

The results from the two naïve raters and the expert raters were considered separately. The intra-class correlation (ICC) was used to determine the level of agreement between the two naïve raters and the two expert raters for interval and ratio variables (i.e. ‘number of players in tackle’ and ‘time taken to leave’). Inter-rater reliability analyses using Cohen’s kappa (*κ*) statistics [17] were used to determine the level of agreement between the two naïve raters and the two expert raters for all other (nominal) variables. Unlike the total percent agreement, Cohen’s kappa considers the proportional agreement that could occur simply by chance. The *κ* coefficients are calculated by considering the proportion of rater agreement and the expected proportion [17]. Using the interpretations of *κ* described by McHugh [34], *κ* agreement was categorized as almost perfect (>.90), strong (.80–.90), moderate (.60–.79), weak (.40–.59), minimal (.21–.39) and none (0–.20). All analyses were performed using IBM SPSS Statistics V.23.0 [21] and used two-sided tests for significance at the 0.05 level, with 95% confidence intervals (CIs).

## Results

The inter-rater reliabilities for the various components of the rating form for both the naïve and expert raters are presented in Table 1. For the naïve raters, 6 of 20 (30%) components of the form, and 5 of 6 (83%) concussions signs, had *κ* values between .60 and .79 (‘moderate’ agreement), while 2 of 2 (100%) interval/ratio variables of the form had very good ICC. According to the interpretations of *κ* described by McHugh [34], 8 of 22 components were categorized as ‘almost perfect’; 3 components were classified as ‘strong’; 2 components were classified as ‘weak’; 2 components were classified as ‘minimal’; and one of the components (playing position) was not classified. For the concussion signs, the naïve raters had no signs that had ‘almost perfect’ or ‘strong’ agreement; 5 (83%) signs were classified as ‘moderate’ and 1 (17%) sign was classified as ‘weak’.

**Table 1:** Inter-rater reliability (*κ*, ICC) for naïve and expert raters for each component of the form

	**Expert raters (** ***κ*** **)**	**McHugh** [34] ***κ*** **Agreement** **Classification**	**Naïve raters** **(** ***κ*** **)**	**McHugh** [34] ***κ*** **Agreement** **Classification**
Component				
Game time	1.00	Perfect	1.00	Perfect
Score	1.00	Perfect	0.95	Perfect
Day/night game	1.00	Perfect	0.70	Moderate
Tackle number	1.00	Perfect	0.75	Moderate
Ball carrier vs. tackler	1.00	Perfect	0.92	Perfect
Playing position	1.00	Perfect	NR	N/A
Tackle height	1.00	Perfect	0.86	Strong
Foul play	0.90	Perfect	0.89	Strong
Offending player on report	0.90	Perfect	1.00	Perfect
Type of play	1.00	Perfect	0.56	Weak
Initial contact	0.90	Perfect	0.90	Perfect
Secondary contact	0.71	Moderate	0.27	Minimal
Region of contact	1.00	Perfect	1.00	Perfect
Location of impact	1.00	Perfect	0.70	Moderate
Anticipation of impact	0.70	Perfect	0.37	Minimal
On-field medical attention	1.00	Perfect	0.65	Moderate
Removal from play	1.00	Perfect	1.00	Perfect
How did the player leave	1.00	Perfect	1.00	Perfect
Location of the field	1.00	Perfect	0.79	Moderate
Did they return to play	1.00	Perfect	0.43	Weak
	**Expert raters** **(ICC)**		**Naïve raters** **(ICC) (95% CIs)**	
Number of players in tackle	1.00		0.87 (0.68–0.95)	
Time taken to leave	1.00		0.99 (0.97–1.00)	
	**Expert raters (** ***κ*** **)** **(95% CIs)**	**McHugh** [34] ***κ*** **Agreement** **Classification**	**Naïve raters (** ***κ*** **)** **(95% CIs)**	**McHugh** [34] ***κ*** **Agreement** **Classification**
Concussion signs				
Clutch or shake head	0.73 (0.46–0.93)	Moderate	0.63 (0.36–0.87)	Moderate
Slow to get up	0.83 (0.15–1.00)	Moderate	0.52 (0.09–1.00)	Strong
Gait ataxia	0.73 (0.47–0.94)	Moderate	0.60 (0.58–0.61)	Moderate
Blank/vacant stare	0.50 (0.23–0.76)	Weak	0.44 (0.15–0.71)	Weak
Unresponsiveness	0.78 (0.56–1.00)	Moderate	0.70 (0.45–0.93)	Moderate
Post-impact seizure	0.65 (N/A)	Moderate	0.53 (0.04–0.90)	Moderate

McHugh *κ* Agreement Classification: almost perfect (>.90), strong (.80–.90), moderate (.60–.79), weak (.40–.59), minimal (.21–.39), and none (0–.20)

*CIs* confidence intervals, *ICC* intra-class correlation, *κ* kappa, *N/A* not applicable, *NR* not recorded

For the expert raters, 19 of 20 (95%) components of the form had *κ* values of between .90–1.00 (‘almost perfect’ agreement) and one (5%) had moderate agreement. The expert raters had perfect ICC for 2 of 2 (100%) interval/ratio variables. For the concussion signs, the expert raters had 1 of 6 (17%) of concussions signs with *κ* values between .80–.90 (‘strong’ agreement); 4 (67%) of were classified as ‘moderate’; and 1 (17%) was classified as ‘weak’ agreement. No signs classified by either the naïve or expert raters had a ‘minimal’ or ‘none’ level of agreement.

There were nine components that were all rated with ‘almost perfect’ agreement by both the naïve and the expert raters (game time, score, whether the concussed player was a ball carrier or a tackler, the number of players involved in the tackle, whether the offending player was placed on report by a match official, the initial contact, the region of contact, whether the player was removed from play, and how the player left the field). The level of agreement between the expert raters and between the naïve raters was also very consistent for the tackle height and whether the injury occurred as a result of foul play (i.e., the offending player was penalized). The naïve raters had a ‘strong’ level of agreement for these components. The naïve raters had a moderate level of agreement on whether the game was played during the night or day, the tackle number in the set, the anatomical location of the impact, and the location of the field where the concussion took place, whereas all of these components had an ‘almost perfect’ level of agreement between the two expert raters. The expert raters also achieved an ‘almost perfect’ level of agreement on the secondary contact, whether the concussed player had anticipated the impact that caused the injury, and the time taken to leave the field of play. However, the naïve raters only had a ‘minimal’ level of agreement on these components. For type of play, and whether or not the player returned to play, the naïve raters had a ‘weak’ agreement on these components compared to the ‘almost perfect’ agreement by the expert raters. Whether or not there was secondary contact was the most difficult component to agree upon; the expert raters’ level of agreement was ‘moderate’ for this component, and the naive raters’ level of agreement was ‘minimal’ (see Table 1).

Regarding concussion signs, slow to get up had the best level of agreement between expert and naïve raters of all possible concussion signs (strong and moderate agreement, respectively), whereas a blank or vacant stare had the worst agreement (both rater groups had a ‘weak’ level of agreement). Clutch or shake head, gait ataxia (or having wobbly legs), unresponsiveness, and post-impact seizure-like features had moderate agreement for both expert and naïve raters.

## Discussion

Rugby League is a full contact collision sport that has high concussion incidence rates [13–15]. The in-game management and decision-making process surrounding concussion is a challenge. Video review is increasingly being used as one method for improving this in-game decision-making process for medical staff, although a standardized approach to the use of such information had not been published. Although there is a large body of research examining on-field markers of concussion and their association with outcome [2–5, 7, 8, 11, 16, 22, 26, 27, 31, 33, 35, 36, 38–40, 42–46], very few of these studies have been focused on possible signs of concussion at the time of injury (versus collected later as part of a questionnaire or interview with the athlete). This study presents a standardized observational form and examines intra-rater and inter-rater agreement on the antecedent events, mechanisms of injury, and concussion signs. Overall, the results of this study suggest that a certain level of knowledge about the game is required to complete the form components accurately. Expert rates achieved an ‘almost perfect’ level of agreement on 21/22 (95%) of components compared to only 9/22 (41%) components for the naïve raters.

In a similar study conducted with the ‘Heads-Up Checklist’ for National Hockey League (NHL) concussions, the naïve raters also had worse agreement across components pertaining to the antecedent events and mechanism of injury compared to the expert raters. Of the 15 components in version 1 of the Heads-Up Checklist, naïve raters 7 (47%) had weak or minimal agreement, compared to only 1 of the 15 (7%) components for the expert raters [20]. For the Heads-Up Checklist, the acceleration of the head (which was not considered a component or review item in our form) was the single component with the worst agreement across naïve and expert raters. Rating secondary contact was also challenging in the hockey study as it was in the current study. The location of the playing surface where the concussion occurred and the time in the game when the concussion occurred were the two components with the strongest agreement by naïve and expert raters for the hockey study [20]. For the current study, the time in the game was rated well. However, the location on the field did not have a high agreement for the naïve raters. The discrepancy between naïve raters for the hockey study compared to this rugby league study may have occurred for at least three reasons. First, we divided the playing surface in our study into 12 different components and the hockey study used fewer zones. Second, the hockey study designated offensive ends and defensive ends, whereas the rugby league study required the raters to record the direction of the play, and some of the disagreement between the naïve raters for the location on the field was due to the indication of the direction of the play. Finally, the hockey study used naïve raters who where more familiar with their sport (i.e., ‘individuals with limited experience who might have played or coached [ice] hockey at a competitive level’), whereas our naïve raters were complete novices, who had limited to no experience even watching the sport as fans and certainly no experience identifying concussions.

In the current study, there were a number of variables that appear to rely on knowledge, understanding, and experience with rugby league match play (i.e., the expert raters outperformed the naïve raters). For example, there were large differences between the coding by expert and naïve raters of variables such as secondary contact and anticipation of impact. There was also a large difference between the coding by expert and naïve raters on whether the player returned to play. This variable required the raters to watch the remainder of a game (following the injury) to determine if the injured athlete subsequently returned to the field of play. Interchanges can occur during play or during a stoppage in play, and they are not always announced on the broadcaster footage. It appears that the naïve raters were not as savvy in identifying the return to play of an interchanged athlete and/or did not identify the athlete as being re-involved in match play following their return to the field of play.

As with our previous video reviews of concussion signs [14, 15], we once again found that determining whether a concussed player had a blank or vacant stare was difficult to agree upon. We had weak agreement between naïve (0.44, 95% CI = 0.15–0.71) and expert (0.50, 95% CI = 0.23–0.76) raters in this study, and our previous work has also revealed difficulty with agreement between raters (i.e., 0.36 (95% CI = 0.29 to 0.43) [14] and 0.62 (95% CI = 0.37 to 0.88) [15]). In a recent Australian Football League (AFL) video review, inter-rater reliability for the blank/vacant stare on first review was reported to be 0.24 (95% CI = 0.04 to 0.41) and minimal improvements were observed on second review [0.26 (95% CI = 0.07 to 0.43)]. The intra-rater reliability in the AFL study was somewhat better for the two raters over the two rating sessions [i.e. 0.63 (95% CI = 0.50 to 0.74) and 0.36 (95% CI = 0.18 to 0.51)]. The concussion sign ‘blank/vacant stare’ was reported to have 9% sensitivity, 100% specificity, 100% positive predictive value and 58% negative predictive value in the sample of AFL concussions [29]. When the quality of the video (including the zoom capacity to see the players face) is limited, attempting to code the presence or absence of a blank or vacant stare from video is challenging [15]. This supports the notion that good-quality video from multiple camera angles are crucial for effective video surveillance of injuries [30]. In the current study, however, this was not a limitation, suggesting that it is also important to have clear definitions, including the inclusion and exclusion criteria for coding concussion signs [30]. In a recent series of video reviews of concussions from the AFL [9, 29, 30], Makdissi and Davis indicated that video review may be an avenue that facilitates the assessment of the mechanism and impact of injury and allows for the identification of brief early signs of concussion [29]. The authors suggest that video analysis may be a useful adjunct to the sideline assessment of possible concussion [29] and that the implementation of a flowchart may improve the timely assessment of concussion [9].

We recently completed a study on the frequency (or base rates) of concussion signs in NRL match play (Gardner et al., under review). That study reviewed every game (*n* = 201) from the 2014 NRL season, which included 127,062 tackles, and found unresponsiveness occurred 52 times [24 (46%) were diagnosed with a concussion], slow to get up occurred 2240 times [60 (3%) were diagnosed with a concussion], clutching or shaking the head occurred 361 times [38 (11%) were diagnosed with a concussion], gait ataxia occurred 102 times [35 (34%) were diagnosed with a concussion], blank or vacant stare occurred 98 times [45 (46%) were diagnosed with a concussion], and a post-impact posturing or seizure occurred 4 times [3 (75%) were diagnosed with a concussion]. The unresponsiveness sign had 40% sensitivity, 91% specificity, 46% positive predictive value, and 89% negative predictive value. The slow to get up sign had 100% sensitivity, 50% specificity, 27% positive predictive value, and 100% negative predictive value. Clutching or shaking the head had 63% sensitivity, 46% specificity, 18% positive predictive value, and 87% negative predictive value. Gait ataxia had 58% sensitivity, 79% specificity, 34% positive predictive value, and 91% negative predictive value. Blank or vacant stare had 75% sensitivity, 84% specificity, 46% positive predictive value, and 95% negative predictive value. Post-impact seizure had 5% sensitivity, 100% specificity, 75% positive predictive value, and 85% negative predictive value in the 2014 NRL season (Gardner et al., under review).

One of the unusual and unexpected findings of this study was the discrepancy observed between the naïve raters in coding variables that were conceivably thought to be obvious (e.g., game time, score, day/night game). The naïve raters did not always have 100% agreement. Because rugby league is a continuous sport, it is common for the game to continue despite an injury, and therefore, the game clock also does not stop. As such, an injury can occur well before the game and the game clock is stopped. The discrepancies in the ‘time in game’ variable are explained by this issue; one of the naïve reviewers recorded the time correctly (i.e., when the injury occurred), whereas the other naïve rater often recorded the time when the game clock was stopped. In terms of the ‘game score’ variable, it is possible that the naïve raters were unfamiliar with teams, and therefore, errors were made in coding the score of each team. For the ‘day/night game’ variable, there were a number of games that were played during twilight, as well as the footage of some of those cases being zoomed in, and the wide view did not make the day/night difference obvious to the naïve raters who do not watch NRL games.

Video review appears to be a useful adjunct to traditional methods for making in-game decisions pertaining to the identification of potential concussion (and an athlete subsequently being removed from play). However, to better understand and quantify the value of this process, future research should be conducted under time limits and/or during a game to replicate the real-world/practical pressure, neither of which was replicated in this study. Future studies might focus on whether agreement between experts improves under ‘ideal circumstances’ (i.e. as many reviews as required without time limitations) versus ‘real-world circumstances’ (i.e. a quick decision required to identify a possible injury and immediately remove the athlete from play).

The current study has several limitations. Firstly, clubs used their own personnel and methods for identifying possible injuries on the field and diagnosing concussions on the sideline, which presumably makes the final specific criteria for a ‘medically diagnosed concussion’ variable across clubs. The current study does not generalize to the real-world use of in-game video analysis because the study was not conducted under the time pressure associated with in-game decision-making. Further, the sample size is small, and only two naïve and two expert reviewers were used. Whether the current results hold true for more cases and a greater number of raters is unknown.

## Conclusions

The present study suggests that determining the presence or absence of a blank or vacant stare is challenging for both naïve and expert raters to rate reliably, but that showing unresponsiveness (i.e. possible LOC), clutching or shaking of the head, a post-impact seizure, or being slow to get up are more reliably rated signs. However, in this study, there was no variability in the clinical outcome measure, as our sample came from a pool of individuals who were all medically diagnosed with a concussion. Therefore, the predictive value of any one component or concussion sign, or a combination of these items, is unknown and may be the focus of future research. Given the variability of in-game decision-making in professional rugby league [13–15], we sought to provide validation of a standardized approach for collecting information surrounding possible concussions to help inform the in-game decision-making process. Although the form was created for all levels of competition, it only had a good level of agreement among experienced raters. Therefore, it might only be useful for those teams or clubs that have experts available to them (i.e., the professional level). For lower levels of competition, the form may have less of a benefit, because the naïve raters had a low level of agreement on many components of the form. It is important to note, however, that the management of suspected concussion at these lower levels should always be conservative. If a concussion is suspected, then the athlete should be removed from play and not returned to play the same day [32]. At the professional level, data collected from this form may allow for a thorough understanding of the situational and contextual factors related to concussion, which may be used to strategize future interventions to reduce the risk of concussion at this level.
